# The Balance Between the Therapeutic Efficacy and Safety of [^177^Lu]Lu-NeoB in a Preclinical Prostate Cancer Model

**DOI:** 10.1007/s11307-023-01851-4

**Published:** 2023-08-28

**Authors:** Marjolein Verhoeven, Joost Haeck, Erik de Blois, Francesca Orlandi, Donato Barbato, Mattia Tedesco, Mark Konijnenberg, Simone U. Dalm

**Affiliations:** 1 Department of Radiology & Nuclear Medicine, Erasmus MC, University Medical Center Rotterdam, 3015 GD Rotterdam, The Netherlands; 2 Advanced Accelerator Applications, a Novartis Company, 10010 Colleretto Giacosa, Italy

**Keywords:** Gastrin-releasing peptide receptor (GRPR), [^177^Lu]Lu-NeoB, Efficacy, Toxicity, Theranostics, Prostate cancer

## Abstract

**Purpose:**

Radiolabeled NeoB is a promising gastrin-releasing peptide receptor (GRPR)–targeting radiopharmaceutical for theranostics of GRPR-expressing malignancies, *e.g.*, prostate cancer (PCa). The aim of this study was to evaluate the effect of different doses of [^177^Lu]Lu-NeoB on the balance between therapeutic efficacy and safety in a preclinical PCa model.

**Procedures:**

To determine the efficacy of [^177^Lu]Lu-NeoB, PC-3 xenografted mice received 3 sham injections (control group) or 3 injections of 30 MBq/300 pmol, 40 MBq/400 pmol, or 60 MBq/600 pmol [^177^Lu]Lu-NeoB (groups 1, 2, and 3, respectively) 1 week apart. To quantify tumor uptake, single-photon emission computed tomography/computed tomography (SPECT/CT) imaging was performed 4 h after the first, second, and third injection on a separate group of animals. For safety evaluations, pancreatic and renal tissues of non-tumor-bearing mice treated with the abovementioned [^177^Lu]Lu-NeoB doses were evaluated 12 and 24 weeks post-treatment.

**Results:**

Treatment of PC-3 tumors with all three studied [^177^Lu]Lu-NeoB doses was effective. Median survival times were significantly (*p* < 0.0001) improved for treatment groups 1, 2, and 3 *versus* the control group (82 days, 89 days, 99 days *versus* 19 days, respectively). However, no significant differences were observed between treatment groups. Quantification of SPECT/CT images showed minimal differences in the average absolute radioactivity uptake, especially after the third injection. Histopathological analysis revealed no clear signs of treatment-related pancreatic toxicity. For the kidneys, atrophy and fibrosis were observed for one animal from group 1 and a chronic inflammatory response was observed for both animals from group 3 at 24 weeks post-treatment.

**Conclusions:**

Treatment with [^177^Lu]Lu-NeoB is effective in a preclinical PCa model. Adjusting the administered dose could positively impact the risk-benefit balance as a higher dose might not lead to an increased therapeutic effect, but it may lead to an increase in toxicological effects in healthy organs such as the kidneys.

**Supplementary Information:**

The online version contains supplementary material available at 10.1007/s11307-023-01851-4.

## Background

The gastrin-releasing peptide receptor (GRPR) has been identified as a promising target for cancer imaging and therapy as it is overexpressed in various solid tumors, *e.g.*, prostate, breast, and gastrointestinal stromal tumors (GIST). GRPR-targeting radiopharmaceuticals can therefore be of theranostic value for these malignancies [1]. Over the years, several GRPR-targeting radiopharmaceuticals have been successfully developed and studied preclinically and clinically [2, 3]. Initial clinical studies have mostly focused on the detection of prostate cancer (PCa) lesions with promising results [4–6]. However, next to the GRPR, overexpression of the prostate-specific membrane antigen (PSMA) has also been reported on PCa cells. In March 2022, the PSMA radiopharmaceutical [^177^Lu]Lu-PSMA-617 was approved by the FDA for the treatment of metastatic castration-resistant PCa following positive results obtained in the VISION trial [7]. As a consequence hereof, multiple studies have focused on identifying whether or not there is a place for GRPR-targeting radiopharmaceuticals alongside PSMA theranostics in PCa management. Taken together, these exploratory studies demonstrated a potential complementary value for PCa patients with both early- and late-stage disease, especially those lacking (sufficient) PSMA expression [8–10]. For example, Baratto *et al*. demonstrated that GRPR-targeted imaging detected 7 additional lesions in 4 patients compared to PSMA-targeted imaging [9]. An additional value of GRPR radiopharmaceuticals may also lie in their combination with alpha emitters that can circumvent severe salivary gland toxicity due to off-target PSMA binding [11].

Despite the above, to date, there is no published research on the therapeutic efficacy of GRPR radiopharmaceuticals labeled with lutetium-177 or other therapeutic radionuclides in PCa patients. Preclinical studies are the basis of clinical translation of radiopharmaceuticals and are a powerful tool to evaluate their therapeutic potential, both in terms of efficacy and safety. The majority of preclinical studies performed with GRPR-targeting radiopharmaceuticals have also focused on PCa and the most commonly used GRPR-positive model is the human PCa cell line PC-3 [12–15]. This includes studies performed by our group exploring the biological characteristics of the potent GRPR-targeting radiopharmaceutical NeoB. NeoB, formerly called NeoBOMB1, is one of the most widely studied radiopharmaceuticals and can be radiolabeled with different radionuclides for theranostic purposes such as gallium-68 for positron emission tomography, indium-111 for single-photon emission tomography (SPECT), and lutetium-177 for peptide receptor radionuclide therapy. Radiolabeled NeoB has been demonstrated to possess a high receptor affinity, high *in vivo* stability, and an excellent tumor targeting capacity [4, 16, 17].

A previous preclinical study evaluated the pharmacokinetic properties and the effect of injected peptide amount on the biodistribution of [^177^Lu]Lu-NeoB in PC-3 xenografted mice [16]. Next to high tumor uptake, significant uptake was observed in the GRPR-expressing pancreas and the kidneys, the latter as a consequence of renal excretion. Fortunately, the radiopharmaceutical was cleared faster from the pancreas than from the tumor. Adjusting the amount of peptide also had a significant impact on pancreatic uptake. The use of a high peptide amount (200 pmol) resulted in a more favorable tumor-to-pancreas ratio, as evidenced by a significantly lower pancreatic uptake and a slightly improved tumor uptake compared to that observed with a low peptide amount (10 pmol). This, together with the fast pancreatic washout, is expected to positively contribute to the therapeutic index of the radiopharmaceutical. However, the therapeutic efficacy of [^177^Lu]Lu-NeoB has not yet been demonstrated in the PC-3 xenografted mouse model.

With the aim of further evaluating NeoB as a theranostic agent for PCa, we determined the effect of different doses of [^177^Lu]Lu-NeoB on the balance between therapeutic efficacy and safety in mice xenografted with PC-3 cells. Since we demonstrated that the injected peptide amount considerably impacts the biodistribution [16], we selected 3 different, but all relatively high, peptide amounts of the radiopharmaceutical using a constant molar activity to potentially increase the absorbed tumor dose: 30 MBq/300 pmol, 40 MBq/400 pmol, and 60 MBq/600 pmol. Next to the efficacy, we performed histopathological analyses on kidney and pancreatic tissues of a small subset of treated non-tumor-bearing animals to determine toxicity of the selected doses of the radiopharmaceutical. Together, this enabled us to determine the effect of different doses on the risk-benefit balance for GRPR-mediated radionuclide therapy with [^177^Lu]Lu-NeoB of PCa.

## Materials and Methods

### Radiolabeling

All chemicals were purchased from Sigma-Aldrich with the highest chemical grade unless otherwise stated. NeoB (Advanced Accelerator Applications, Saint-Genis-Pouilly, France) was radiolabeled with lutetium-177 (IDB Holland, Baarle-Nassau, The Netherlands) with a molar activity of 100 MBq/nmol according to the following steps. Three solutions were prepared prior to radiolabeling: solution 1: 0.250 mL gentisic acid (20 mg/mL) + 1.75 mL acetate buffer (0.5 M, pH 4.5–5.0); solution 2: ~ 1 mg DOTA-NeoB + 5 mL of the non-ionic surfactant Kolliphor HS 15 (Merck KGaA, Darmstadt, Germany) (2 mg/mL); solution 3: 3 mL ascorbic acid (22.5 mg/mL) + 1 mL acetate buffer (1 M, pH 5). For radiolabeling, 0.5 mL of lutetium-177 (50 GBq/mL) was added to 1 mL of solution 1. Subsequently, ~ 66.2 μL of solution 2 was added, and the mixture was left in a 95 °C dry bath for 7 min. Hereafter, the product was left to cool down for 5 min, and 0.5 mL of solution 3, 0.42 mL MilliQ, and 50 μL of diethylenetriaminepentaacetic acid (DTPA) (4 mM) were added.

The radiochemical yield (RCY) and the radiochemical purity (RCP) of [^177^Lu]Lu-NeoB were measured to determine the quality of labeling. The RCY was measured by instant thin-layer chromatography on silica gel (Varian, Houten, The Netherlands) using a 1.0 M aqueous solution of ammonium acetate:methanol (40:60 v/v) as the mobile phase (Table S1). The RCP was measured by high-pressure liquid chromatography (HPLC) using an Alliance HPLC-system (Waters, Etten-Leur, The Netherlands) containing the W2487 Waters Dual λ Absorbance UV Detector (Figure S1). UV absorbance was measured at 278 nm and a peptide XB-C18 column (3.6 μm, 150 × 4.6 mm, Phenomenex B.V., Utrecht, The Netherlands) was used with a gradient profile of 0.1% formic acid and acetonitrile at 1 mL/min (Table S2). Radioactivity was monitored with a system holding a NaI detector, digital multichannel analyzer, and dedicated software (MetorX BV, Goedereede, The Netherlands), connected to the HPLC-system. The RCY and RCP of each labeling are listed in Table S3. In 2/12 cases, the RCP was less than 85% as a consequence of non-incorporated lutetium-177, which was complexed with DTPA after labeling to prevent bone uptake [18]. In both cases, the injected dose was corrected to obtain the desired peptide mass per administration. After labeling, [^177^Lu]Lu-NeoB was diluted in PBS (Gibco, Paisley, UK) + 60 ppm Kolliphor HS 15 (3 mg/50 mL) to reach the desired concentrations.

### *In Vivo *Efficacy Study

All animal studies were conducted in agreement with the Animal Welfare Committee requirements of the Erasmus MC and in accordance with accepted guidelines. PC-3 cells (ATCC, Manassas, VA, USA) were cultured in Ham’s F-12K (Kaighn’s) medium (Gibco) supplemented with 10% fetal bovine serum, penicillin (100 units/mL), and streptomycin (100 μg/mL) at 37 °C in a 5% CO_2_ atmosphere. Six- to eight-week-old male balb c nu/nu mice (Janvier Labs, Le Genest-Saint-Isle, France) were subcutaneously inoculated on the right flank with 4 × 10^6^ PC-3 cells in 200 μL inoculation medium (1/3 Matrigel high concentration (Corning, Corning, NY, USA) + 2/3 Hank’s balanced salt solution (Thermofisher Scientific, Waltham, MA, USA)).

Four weeks post-tumor cell inoculation, when an average tumor size of 543 ± 177 mm^3^ was reached, animals (*n* = 55) were divided into four groups: control group (*n* = 10) and therapy group 1–3 (*n* = 15 per group). Animals of the control group received 3 consecutive sham injections, and animals belonging to group 1, 2, and 3 received 3 consecutive injections of 30 MBq/300 pmol [^177^Lu]Lu-NeoB, 3 injections of 40 MBq/400 pmol [^177^Lu]Lu-NeoB, or 3 injections of 60 MBq/600 pmol [^177^Lu]Lu-NeoB, respectively, while under isoflurane/O_2_ anesthesia. The total volume of each injection was 200 μL, the injections were administered intravenously in the tail vein, and injections were given 1 week apart. Animal weight and tumor size were measured twice a week. If tumor volume was ≥ 2000 mm^3^, a decrease in animal weight of ≥ 20% in relation to the weight at the start of the experiment or a decrease in weight of > 10% within 48 h was observed (humane endpoint criteria predetermined at our institute), animals were removed from the study. Animals were followed until the maximum allowed age of 230 days was reached.

### *In Vivo *Toxicity Study

A preliminary toxicity assessment was conducted evaluating the effect of treatment on the pancreas and kidneys. For this, 6–8-week-old non-tumor-bearing balb c nu/nu male mice (*n* = 16, Janvier Labs) received the same treatment as the animals included in the efficacy study. At 2 different time points after the last therapeutic injection, *i.e.*, 12 weeks and 24 weeks p.i. (*n* = 2 per time point for each group), animals were euthanized and pancreatic and renal tissues were collected for pathological analysis. During the toxicity study, animals were weighed twice a week and monitored for the aforementioned humane endpoints.

### SPECT/CT Imaging

To quantify tumor uptake, SPECT/computed tomography (CT) imaging was performed in an additional group of PC-3 xenografted animals (*n* = 6; *n* = 2 per group). When tumor size was 477 ± 57 mm^3^, animals were injected with 3 consecutive injections of 30 MBq/300 pmol, 40 MBq/400 pmol, or 60 MBq/600 pmol [^177^Lu]Lu-NeoB. The total volume, injection route, and sequence of radiopharmaceutical administration were similar to the efficacy study. Four hours after the first, second, and third therapeutic injection, whole-body SPECT/CT scans were performed on a hybrid SPECT/CT scanner (VECTor5, MILabs, Utrecht, The Netherlands) while animals were under 1.5–2% isoflurane/O_2_ anesthesia. SPECT was performed in 30 min with 40 bed positions using a 2.0-mm pinhole collimator with a reported spatial resolution of 0.85 mm [19]. SPECT images were reconstructed using photopeak energy windows with 113 and 208 keV centers with a 20% background window on either side of the photopeak. The SROSEM reconstruction method was used with a voxel size of 0.8 mm^3^ [20]. Furthermore, a post-reconstruction 3-dimensional Gaussian filter was applied (1-mm full width half maximum) to the registered SPECT images. CT was performed with the following settings: 0.24 mA, 50 kV, full angle scan, 1 position. The CT was reconstructed at 100 μm^3^. Registered SPECT/CT images were analyzed in PMOD software (PMOD 3.9, Zurich, Switzerland). CT images were used to manually draw a volume of interest (VOI) around the tumor to obtain the tumor volume and then the corresponding total SPECT signal in this VOI. The activity correction factor was determined by scanning an Eppendorf tube filled with a known amount of radioactivity and processing it with the same reconstruction settings as for the animals.

### Pathological Analysis

Pancreatic and renal tissues collected for pathological analysis were formalin fixed and paraffin embedded. To determine differences in tissue structure between the 4 groups, hematoxylin and eosin (H&E) staining was performed on 4-μm-thick tissue slices using the Ventana Symphony™ H&E protocol (Ventana Medical Systems, Tucson, AZ, USA). In total, 3 tissue slices, 50 μm apart from each other, of the pancreas and kidney tissue of each animal were evaluated by experienced pathologists.

### Tumor Growth Delay Analysis

The tumor doubling times were determined by fitting an exponential growth function to the tumor volume over time in the control group. In the therapy groups, an interval with exponential tumor volume decline was fitted with onset of exponential regrowth after the nadir time. The growth curves were extrapolated beyond the censoring time points for mice with tumors that exceeded the maximum allowable size (> 2000 mm^3^) to determine overall average growth statistics. Tumor growth delay time was individually determined and defined as the time needed to reach the maximum tumor size of 2000 mm^3^. Animals that presented with a tumor of which the tumor was still shrinking at the end of the study or when the animal had to be removed from the study (*e.g.*, early sacrifice due to reasons specified in the “Results” section) were not included in this analysis. Additionally, animals with complete remission were included for determination of the median tumor growth delay time.

### Statistics

Prism software (version 5.01, GraphPad Software, San Diego, CA, USA) was used for statistical analyses. *P* values of < 0.05 were considered statistically significant. The difference in tumor volume and tumor growth delay times for the 4 groups was analyzed using one-way ANOVA with Bonferroni’s multiple comparison test. Curve fitting to extrapolated individual data was performed according to the least-squares fit with the Pearson *R*^2^ to quantify its goodness. The log-rank test was used to perform statistical analysis of survival data.

## Results

### Treatment Efficacy

**Fig. 1:**
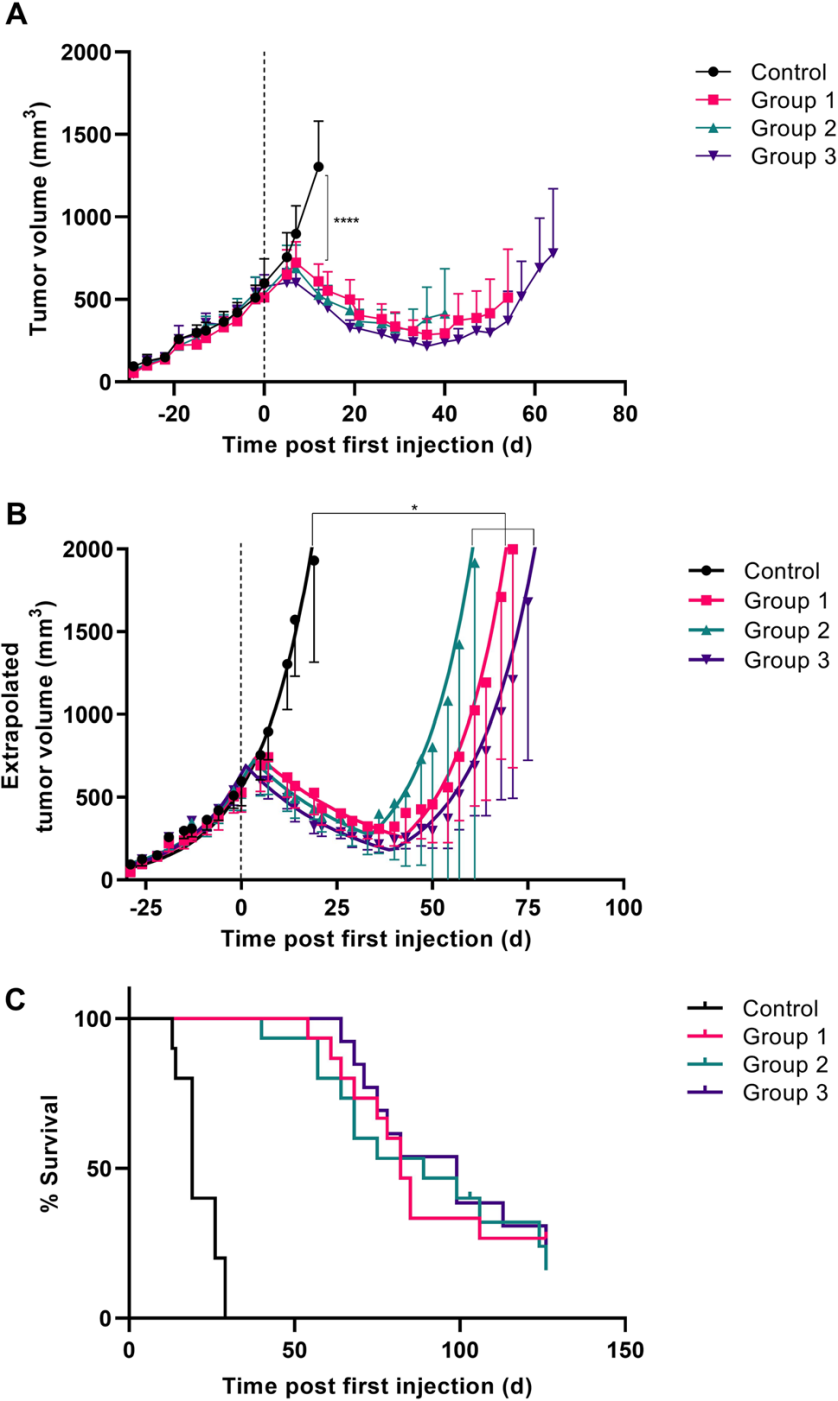
Tumor volume (**A**), extrapolated tumor volume (**B**), and percent of survival (**C**) of PC-3 tumor-bearing mice untreated/sham-treated (control), or treated with 3 injections of 30 MBq/300 pmol (group 1), 40 MBq/400 pmol (group 2), or 60 MBq/600 pmol of [^177^Lu]Lu-NeoB (group 3) on day 0, 7, and 14. The mean tumor volume with 95% confidence interval is presented for the time period that all data points of the group were available (*i.e.*, tumor volume of no animal in the group exceeded 2000 mm^3^) (**A**) or for the duration until the tumor limit of 2000 mm^3^ was reached based on extrapolated growth curves (**B**). The dotted line indicates the start of treatment. *p < 0.05, ****p < 0.0001

Therapy with all three different doses of [^177^Lu]Lu-NeoB was effective; a significant difference in tumor volume between the control group and treatment groups was observed. At day 12 after the start of treatment, mean tumor volumes were 1305 ± 385 mm^3^ for the control group and 610 ± 190 mm^3^, 528 ± 162 mm^3^, and 495 ± 106 mm^3^ for groups 1, 2, and 3, respectively (*p* < 0.0001) (Fig. 1A). Initial tumor shrinkage was followed by regrowth for all treatment groups. Using extrapolated data, a significant difference in tumor growth delay time was observed; the animals in the control group reached a tumor size of 2000 mm^3^ in a median of 17 days, while this was 82 days, 85 days, and 89 days for group 1, group 2, and group 3, respectively (*p* < 0.05) (Fig. 1B). Interestingly, there was no significant difference in tumor growth delay times between the treatment groups despite the different administered doses.

In line with the above, animals in the treatment groups had a significantly improved survival rate compared to those in the control group (*p* < 0.0001) (Fig. 1C). Median survival times were 19 days, 82 days, 89 days, and 99 days for the control group, group 1, group 2, and group 3, respectively. In addition, 2 animals from group 1 and 1 animal from group 2 did not show any tumor regrowth after a complete response (individual growth curves are presented in Figure S2).

In total, two animals from group 3 were excluded from the efficacy study because of the following reasons: 1 animal was found dead after the first injection and 1 animal had a very small tumor at the start of therapy (outlier based on Grubb’s test). The majority of animals were removed from the study due to the exceeding maximum allowable tumor size of 2000 mm^3^ (study endpoint criteria). However, two animals (from group 2) had to be removed for a different reason: 1 animal had more than 20% weight loss relative to its weight at the start of therapy and 1 animal presented fluid build-up in the peritoneal cavity. It remains unclear whether these events were related to treatment.

### SPECT/CT Imaging

**Fig. 2:**
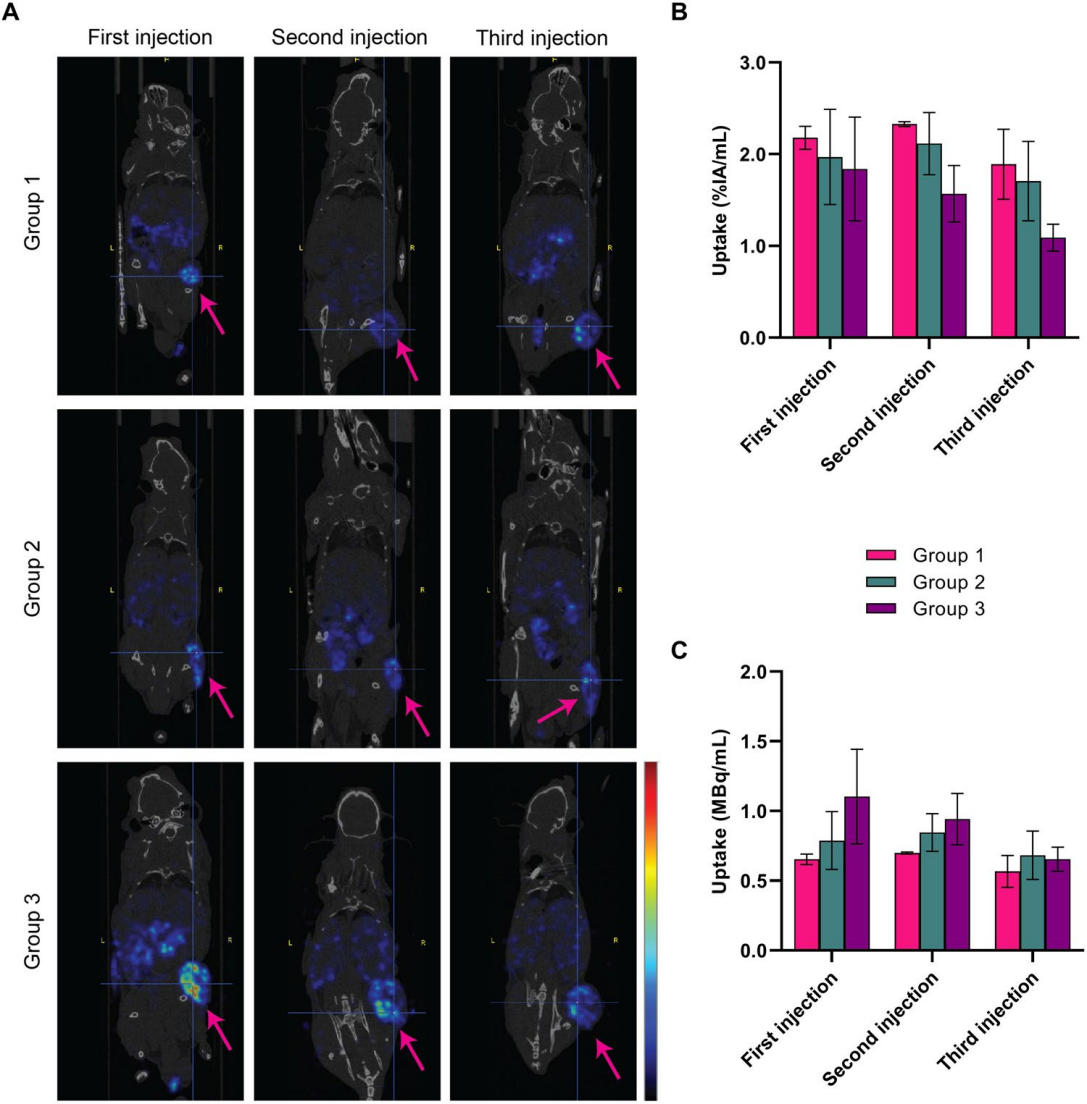
Representative SPECT/CT images (**A**) acquired 4 h post the first, second, and third injection of animals treated with 3 × 30 MBq/300 pmol (group 1), 3 × 40 MBq/400 pmol (group 2), or 3 × 60 MBq/600 pmol of [^177^Lu]Lu-NeoB (group 3). Images show an overlay of a CT slice and the corresponding SPECT slice on which the cross-section of the tumor is clearly visible. Arrows indicate the tumor and the scale bar shows the linear scaling running from min-max of the signal present in the images. The mean quantified tumor uptake with the range is expressed as %IA/mL (**B**) and MBq/mL (**C**) (*n* = 2 per group)

Representative SPECT/CT images obtained 4 h post each administered injection of 30 MBq/300 pmol, 40 MBq/400 pmol, and 60 MBq/600 pmol of [^177^Lu]Lu-NeoB are depicted in Fig. 2A. The highest quantified relative tumor uptake, expressed as percentage of the injected activity per milliliter (%IA/mL), was observed in group 1 (Fig. 2B). However, after the first and second injection, the average absolute radioactivity uptake was highest for group 3, followed by group 2 and group 1 (Fig. 2C). Furthermore, no clear differences in tumor uptake were observed between repeated injections.

### Toxicity

**Fig. 3:**
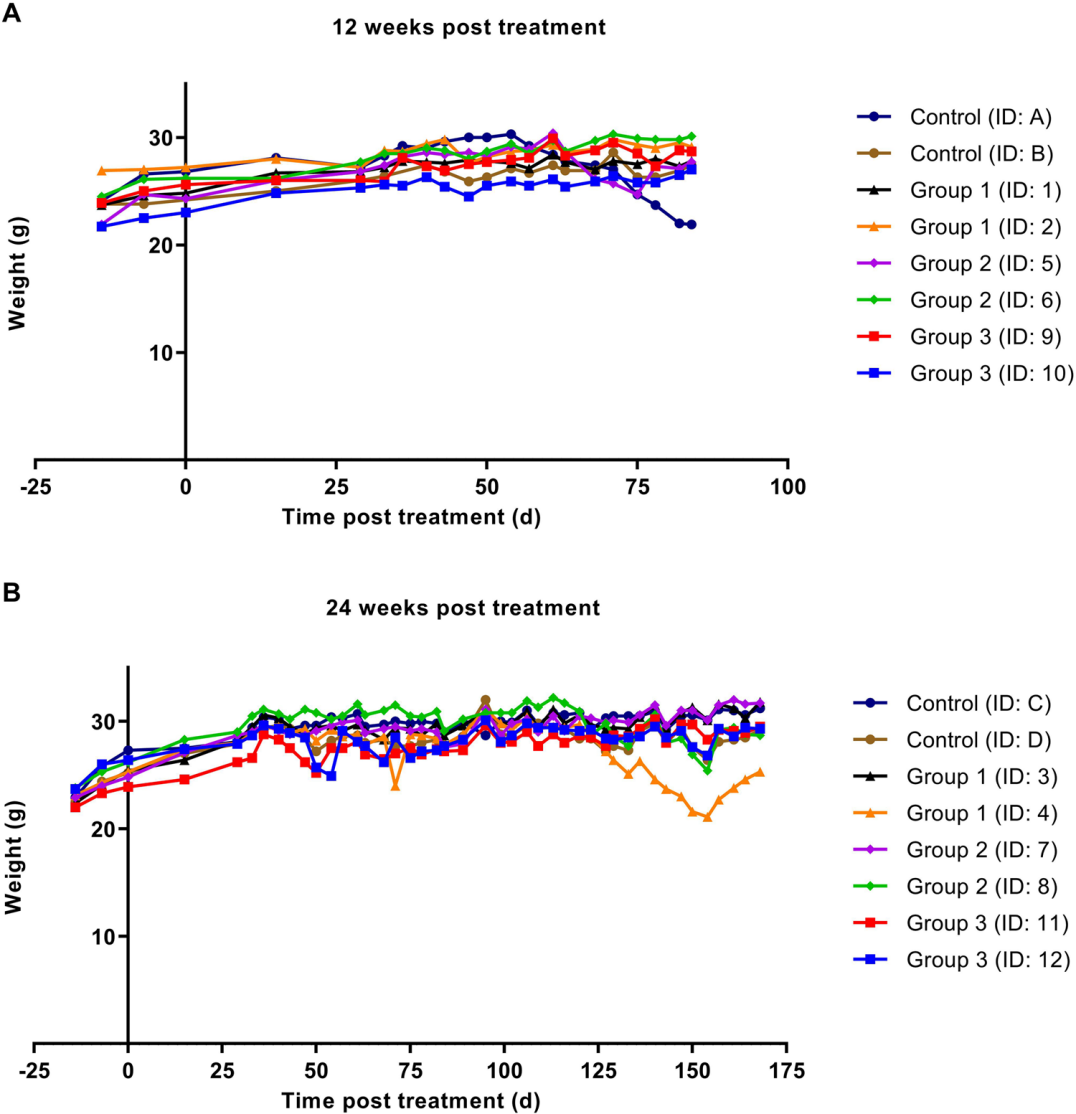
Animal weight before and after treatment with 3 × sham injection (control) or 3 × 30 MBq/300 pmol (group 1), 3 × 40 MBq/400 pmol (group 2), and 3 × 60 MBq/600 pmol of [^177^Lu]Lu-NeoB (group 3) up to 12 weeks (**A**) and 24 weeks (**B**). ID numbers represent the identification number for each individual animal

To assess whether the three increasing activity doses administered resulted in toxicity, animal body weight was monitored and pancreatic and renal tissues excised 12 and 24 weeks post-treatment were analyzed. No critical decrease in weight loss was observed in animals included in the toxicity study throughout the follow-up period (Fig. 3). Animal weight increased during the first weeks and stayed relatively stable over time. One animal in the control group (ID: A) and 1 animal from group 1 (ID: 4) showed a decrease in weight, but this was less than 10% within 48 h.

**Fig. 4:**
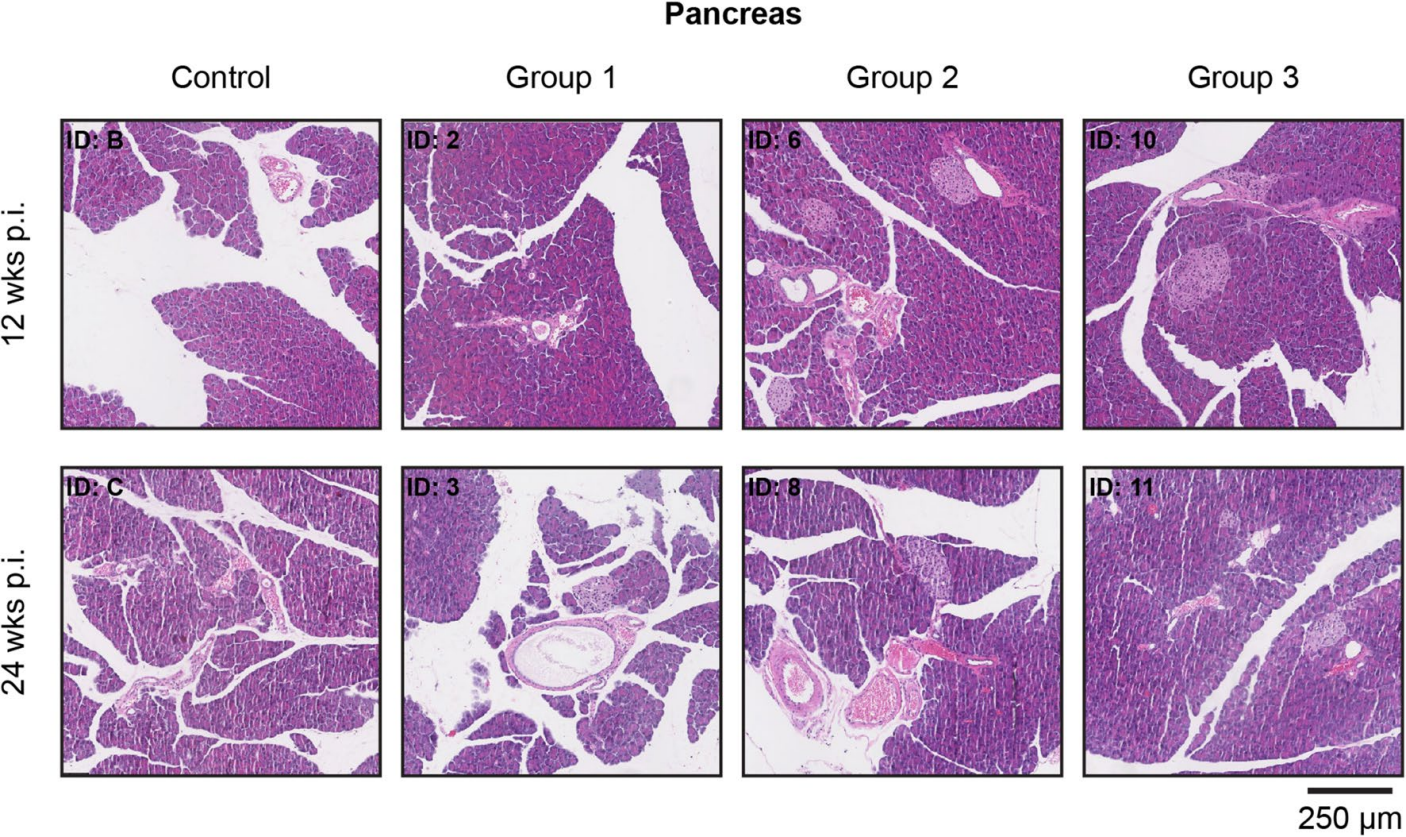
Representative H&E staining of pancreatic tissue from untreated (control) and treated animals (3× 30 MBq/300 pmol (group 1), 3× 40 MBq/400 pmol (group 2), and 3× 60 MBq/600 pmol of [^177^Lu]Lu-NeoB (group 3)) after 12 and 24 weeks. ID numbers represent the identification number of the animal

**Fig. 5:**
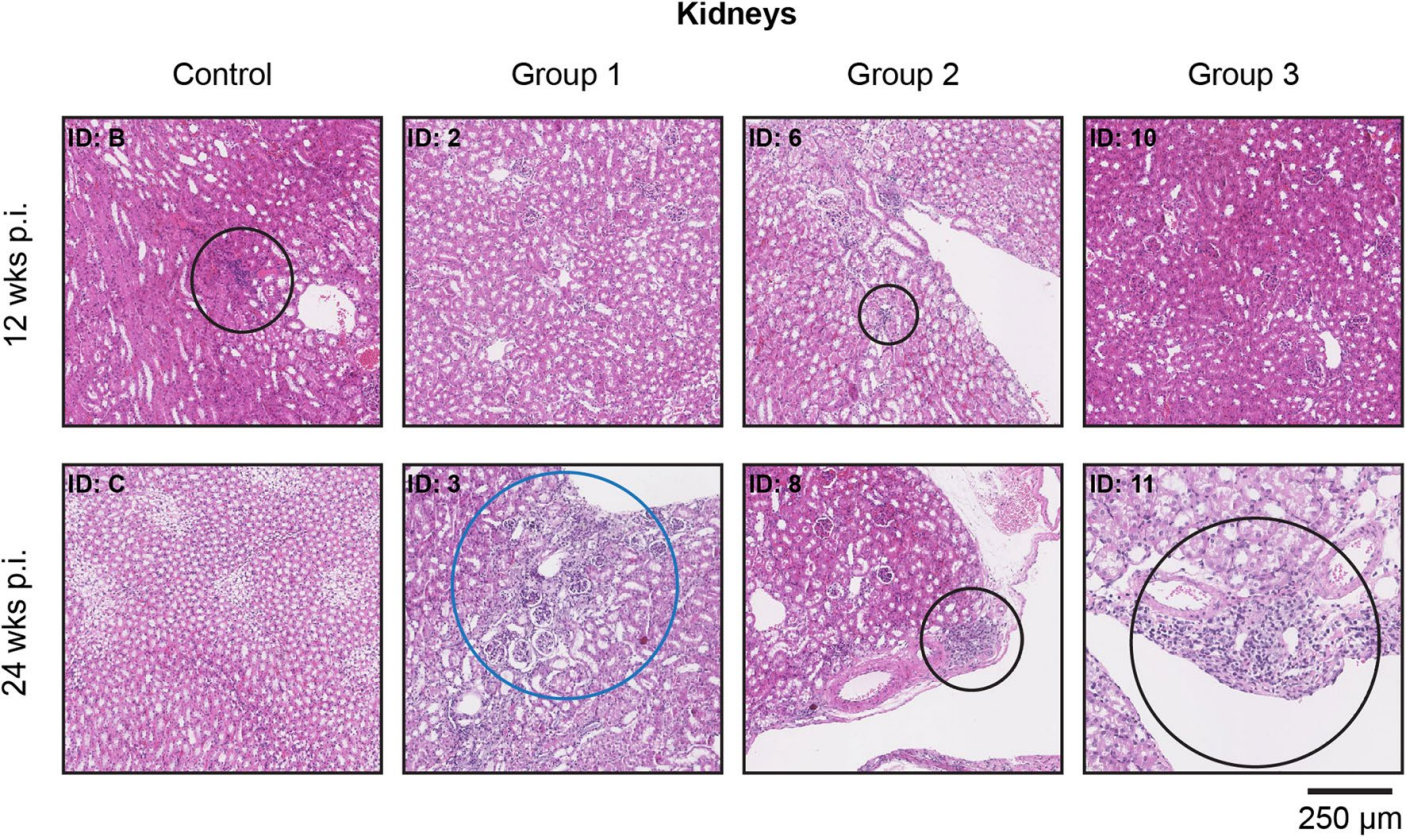
Representative H&E staining of renal tissue from untreated (control) and treated animals (3× 30 MBq/300 pmol (group 1), 3× 40 MBq/400 pmol (group 2), and 3× 60 MBq/600 pmol of [^177^Lu]Lu-NeoB (group 3)) after 12 and 24 weeks. Areas circled in black indicate regions with low (ID: B, 6 and 8) or high (ID: 11) lymphocyte infiltration. The area circled in blue shows a region of atrophy and fibrosis (ID: 3). ID numbers represent the identification number of the animal

Histopathological analysis revealed (very) small areas of lymphocyte infiltration in pancreatic tissues 24 weeks post-treatment (*n* = 4: 1/2 control group; 1/2 group 1; 1/2 group 2; 1/2 group 3) (Fig. 4). Concerning the kidneys, small areas with lymphocyte infiltration were observed at 12 weeks (*n* = 3: 1/2 control group; 2/2 group 2) and 24 weeks (*n* = 2: 1/2 group 1; 1/2 group 2) after the last therapeutic injection. Representative images are depicted in Fig. 5. Since the abovementioned low level of infection was observed in the pancreas and kidneys of both control and treated animals, this finding was considered unrelated to therapy. However, a possible activity dose-related effect was found in the kidneys of both animals from group 3 that were euthanized 24 weeks after therapy. Here a chronic inflammatory response was observed. Furthermore, 24 weeks after therapy, atrophy and fibrosis were observed in the kidneys of 1 animal receiving the lowest therapeutic dose (ID: 3).

## Discussion

Radiolabeled NeoB is considered a promising theranostic agent for various high-incidence solid cancers, including PCa. Initial imaging studies with [^68^Ga]Ga-NeoB have shown potential for the detection of both GRPR-expressing primary and metastatic lesions in PCa patients [4]. Importantly, a number of researchers have suggested that GRPR theranostics could complement the use of PSMA radiopharmaceuticals in PCa management. However, the potential of [^177^Lu]Lu-NeoB for therapeutic purposes in PCa specifically has not yet been established. The present study was designed to determine the effect of different doses of [^177^Lu]Lu-NeoB on the balance between therapeutic efficacy and safety in the most widely studied GRPR-positive preclinical PCa model for GRPR theranostics. To this end, we selected 30 MBq/300 pmol, 40 MBq/400 pmol, and 60 MBq/600 pmol of [^177^Lu]Lu-NeoB for evaluation, which we hypothesized would lead to favorable tumor-to-organ ratios based on previous results demonstrating the importance of peptide amount for an optimal biodistribution of [^177^Lu]Lu-NeoB [16].

Regarding efficacy, this study demonstrated a significant delay in tumor growth and an increased median survival in all treatment groups *versus* the control group. These results match those of other lutetium-177-labeled GRPR antagonists, *i.e.*, SB3, RM2, and gluBBN, tested in a preclinical setting using the same PCa model [21–23]. Utilizing mice bearing gastrointestinal stromal tumors, Montemagno *et al*. [24] recently also showed the potency of [^177^Lu]Lu-NeoB. Although a direct comparison of our findings with the aforementioned studies is hampered by the differences in treatment schedules, administered doses, and/or choice of tumor model, all of these studies underline the potential of GRPR-targeting radionuclide therapy. Additionally, previous studies with other GRPR-targeting antagonists have shown that this effect is not due to the peptide itself, but rather an effect of the radioactivity delivered to the tumors via the peptide [21, 25].

Our study design also allowed us to identify the effect of various administered doses on the therapeutic efficacy. Surprisingly, no significant differences were found between the three treatment groups receiving 3 × 30 MBq/300 pmol, 3 × 40 MBq/400 pmol, or 3 × 60 MBq/600 pmol of [^177^Lu]Lu-NeoB at a 1-week interval. Imaging studies were used to support this finding by evaluating tumor uptake. While this study was only conducted in two mice, the imaging data suggests that a level of receptor saturation might occur when higher peptide amounts are administered. A slightly lower relative tumor uptake was observed with increasing peptide amounts. Further research is required to confirm and validate these findings on receptor saturation, for example, by performing an *in vivo* blocking study with different peptide amounts.

Quantified absolute tumor uptake on SPECT/CT images after the first and second injection still showed a trend with uptake being highest in group 3 and lowest in group 1. However, this trend was not observed after the third injection. These results suggest that the differences in absolute activity between the groups and thus the absorbed dose administered to the tumors were probably too small to see a difference in therapeutic effect. Our findings appear to be consistent with another study that found minimal differences in response and survival between animals treated with a total of 72 or 144 MBq [^177^Lu]Lu-RM2 administered at repeated doses of 12 MBq/200 pmol and 24 MBq/400 pmol [23]. The authors of the respective paper hold the view that this may be due to the non-stochastic effects of radiation. However, our preliminary findings suggest that receptor saturation, at least partly, might play a role under these experimental conditions.

To assess toxicity, we conducted a separate small study in non-tumor-bearing animals to study relatively long-term effects on the pancreas and kidneys for these specific doses. In this initial toxicity evaluation, we specifically selected the two organs that presented the highest uptake and are therefore expected to be dose-limiting for [^177^Lu]Lu-NeoB treatment. Mice tolerated doses up to 3 × 60 MBq/600 pmol of [^177^Lu]Lu-NeoB, with no signs of weight loss or other changes that could possibly be associated with treatment. Despite the known high [^177^Lu]Lu-NeoB uptake in the GRPR-expressing pancreas, we found no treatment-related signs of pancreatic toxicity in the H&E-stained sections. The radiation dose to which the pancreas is exposed may be limited due to lower pancreatic uptake associated with higher peptide amounts and the relatively rapid [^177^Lu]Lu-NeoB clearance from this organ [16]. In accordance with our findings, multiple studies have shown that no serious pancreatic side effects are to be expected from treatment with radiolabeled GRPR antagonists [23, 24, 26, 27]. In our follow-up study, we performed extensive toxicological evaluations including the assessment of blood parameters. In line with the current study, we found no indications of pancreatic toxicity [26].

NeoB, like other radiopharmaceuticals, is primarily cleared from the body via the kidneys. Interestingly, at week 24, we found a chronic inflammatory response in the kidneys of both animals receiving the highest peptide amount. In addition, atrophy and fibrosis were reported for one animal of treatment group 1. Since we found no histopathological abnormalities after 12 weeks, these findings may suggest that renal toxicity is a late effect. This result has not previously been reported for other radiolabeled GRPR antagonists, most likely because the kidneys were histologically assessed at the endpoint of therapy studies, which is often around 12 weeks. Although our results should be interpreted with caution due to the small sample size, the initial observations suggest that there may be a link between the extent of toxicity and radioactivity dose injected. Our recently published work, previously mentioned, also reported late kidney damage at 19 and 43 weeks after treatment with high doses of 3 × 80 MBq/800 pmol or 3 × 120 MBq/1200 pmol [^177^Lu]Lu-NeoB. Consistent with the implications of our current study, our previous study established a dose-effect relationship. Of note, 3 consecutive injections of a high peptide amount (1200 pmol) NeoB were also evaluated in this study demonstrating that the peptide labeled with non-radioactive lutetium-175 does not cause organ toxicity [26].

Overall, the results of this investigation have shown that [^177^Lu]Lu-NeoB is a promising treatment option for PCa and that administration of a higher dose does not automatically lead to an increased therapeutic effect, possibly due to receptor saturation. It may, on the other hand, lead to an increase in toxicological effects in the kidneys. Thus, adjusting the dose could positively impact the balance between efficacy and safety. However, the observed kidney damage needs to be interpreted with caution as it cannot be directly extrapolated to patients. Among others, the size difference between human and mouse kidneys should be taken into account. In humans, treatment with lutetium-177-labeled agents most likely leads to a more heterogeneous distribution of the radioactivity dose.

## Conclusions

Treatment with [^177^Lu]Lu-NeoB is effective in a preclinical PCa model. While a higher dose may not significantly affect therapeutic efficacy possibly due to receptor saturation, it may adversely affect safety. These findings contribute to our understanding of the effect of administered dose on the risk-benefit balance of GRPR-mediated radionuclide therapy with [^177^Lu]Lu-NeoB and provide a basis for clinical translation of this radiopharmaceutical.

### Supplementary Information

ESM 1 (DOCX 1.07 MB)

## Data Availability

Data is available from the corresponding author on reasonable request.
