# Supplementary material for: The Balance Between the Therapeutic Efficacy and Safety of [177Lu]Lu-NeoB in a Preclinical Prostate Cancer Model
Source: Mol Imaging Biol. 2023 Aug 28;26(1):114–23. doi: 10.1007/s11307-023-01851-4 (PMC10828073; doi:10.1007/s11307-023-01851-4)
Supplement: Supplementary file 1 — (DOCX 1.07 MB) [file 11307_2023_1851_MOESM1_ESM.docx]

**Electronic Supplementary Material**

*Article title*: The balance between the therapeutic efficacy and safety of [^177^Lu]Lu-NeoB in a preclinical prostate cancer model
*Journal*: Molecular Imaging and Biology
*Author names and affiliations*: Marjolein Verhoeven^1^, Joost Haeck^1^, Erik de Blois^1^, Francesca Orlandi^2^, Donato Barbato^2^, Mattia Tedesco^2^, Mark Konijnenberg^1^ and Simone U. Dalm^1,*^

^1^ Erasmus MC, University Medical Center Rotterdam, Department of Radiology and Nuclear Medicine, 3015 GD Rotterdam, The Netherlands
^2^ Advanced Accelerator Applications, a Novartis company, Colleretto Giacosa 10010, Italy

^*^ Corresponding Author: Simone U. Dalm; s.dalm@erasmusmc.nl; +31107035781

*Table S1.* Values of a representative instant thin-layer chromatogram using a mobile phase of NH_4_Ac/MeOH (40/60).

| **Counts (CPM)** | **Peak (%)** |
| --- | --- |
| 4 | 0.55 |
| 712 | 98.07 |
| 4 | 0.55 |
| 4 | 0.55 |
| 2 | 0.28 |

*Table S2.* Gradient profile used for HPLC.

| **Time (min)** | **0.1% formic acid (%)** | **Acetonitrile (%)** |
| --- | --- | --- |
| 0 – 2 | 85 | 15 |
| 2 – 9 | 60 | 40 |
| 9 – 11 | 60 | 40 |
| 11 – 11.5 | 0 | 100 |
| 11.5 – 13 | 0 | 100 |
| 13 – 13.1 | 85 | 15 |
| 13.1 – 15.5 | 85 | 15 |

*Table S3.* Radiochemical yield and radiochemical purity of radiolabeling.

|  |  | **Radiochemical yield (%)** | | **Radiochemical purity (%)** | |
| --- | --- | --- | --- | --- | --- |
| **Efficacy studies** | First injection  t=0 days | >99 | >99 | 96.0 | 96.1 |
|  | Second injection  t=7 days | >99 | >99 | 96.4 | 95.5 |
|  | Third injection  t=14 days | 93.0 | >99 | 89.2 | 97.0 |
| **Imaging studies** | First injection  t=0 days | >99 | | 94.7 | |
|  | Second injection  t=7 days | 93.8 | | 67.0 | |
|  | Third injection  t=14 days | 92.7 | | 78.5 | |
| **Toxicity studies** | First injection  t=0 days | >99 | | 98.5 | |
|  | Second injection  t=7 days | >99 | | 98.8 | |
|  | Third injection  t=14 days | >99 | | 99.3 | |


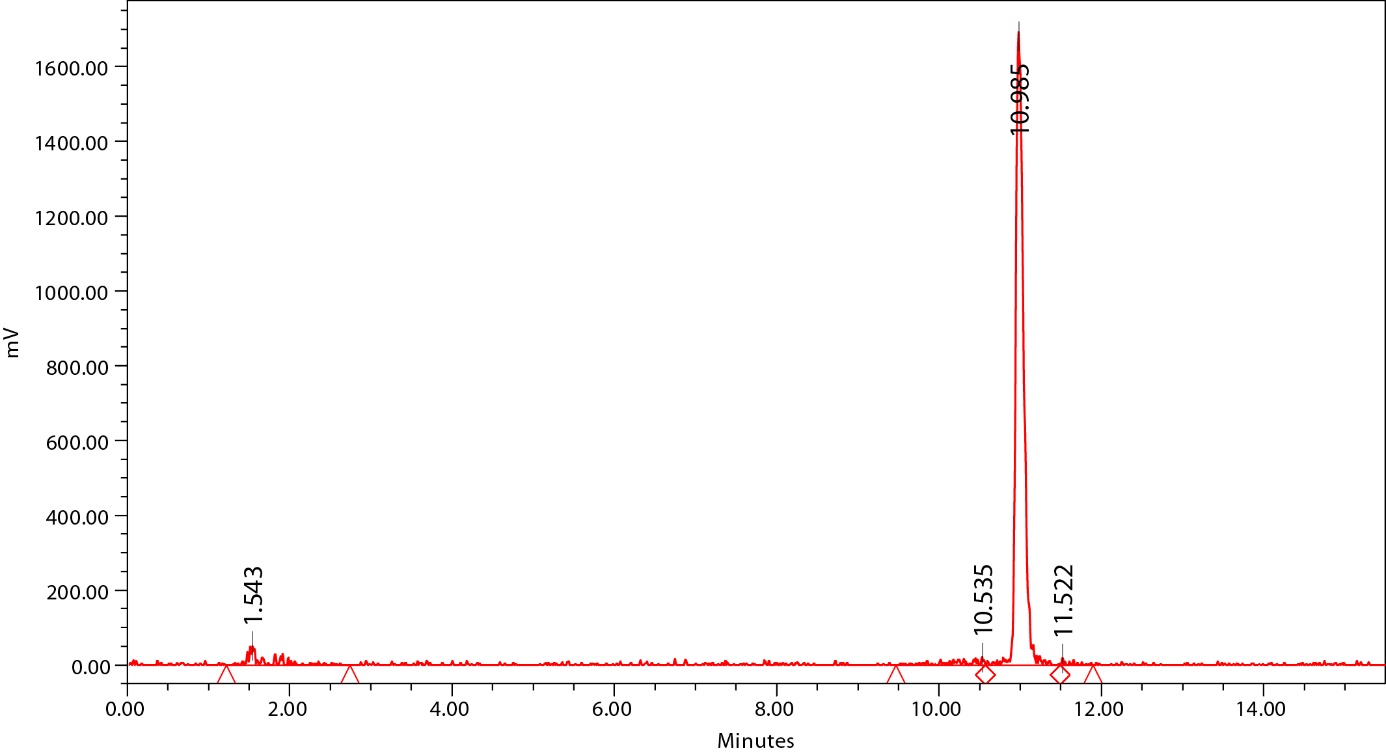
*Figure S1.* Representative HPLC chromatogram of [^177^Lu]Lu-NeoB labeling.


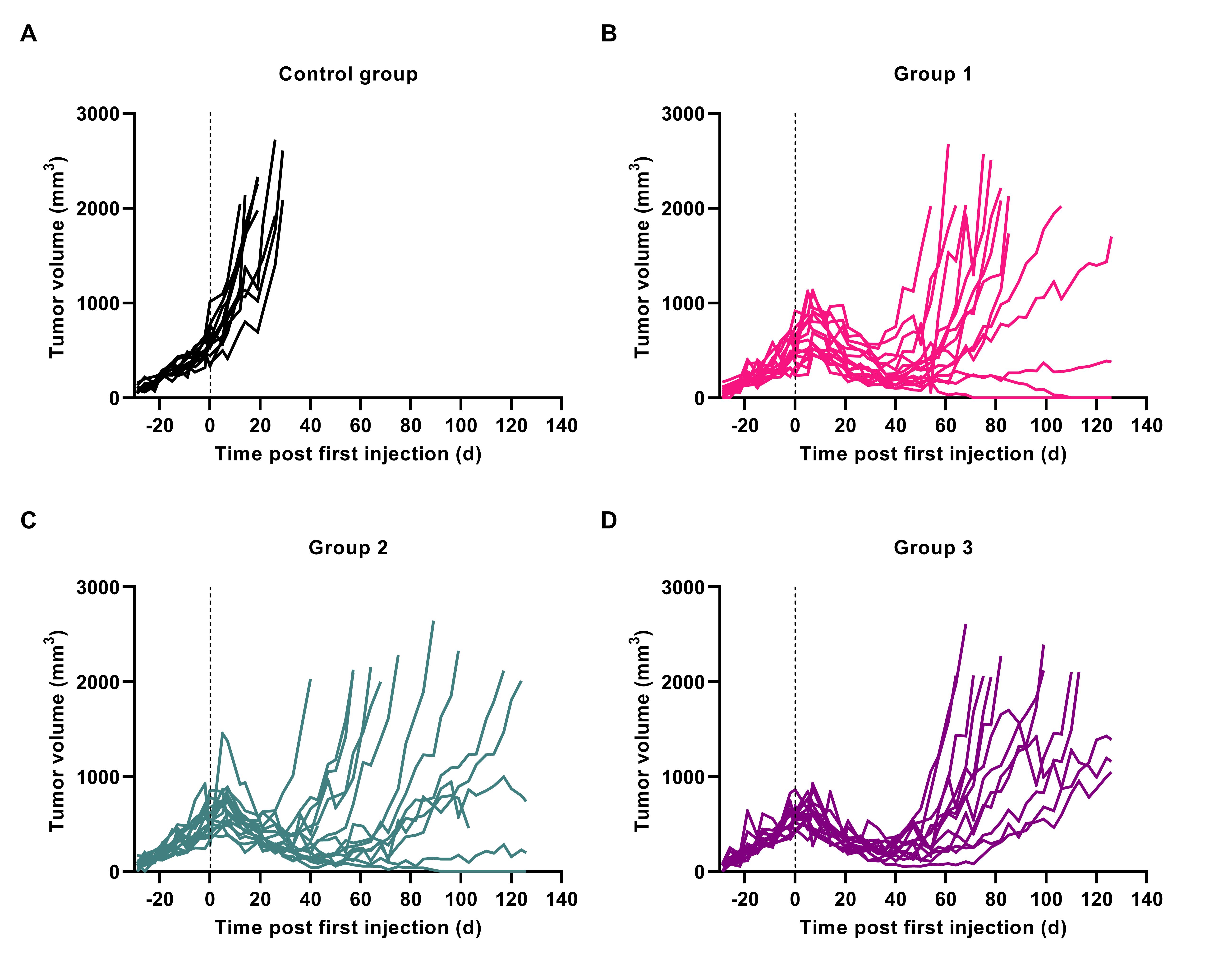


*Figure S2.* Individual tumor growth curves of animals (a) untreated (control; n=10) or treated with 3 injections of (b) 30 MBq/300 pmol (group 1; n=15), (d) 40 MBq/400 pmol (group 2; n=13) or (d) 60 MBq/600 pmol of [^177^Lu]Lu-NeoB (group 3; n=13) on day 0, 7 and 14. The dotted line indicates the start of treatment.
